# Circulating Soluble ACE2 and Upstream microRNA Expressions in Serum of Type 2 Diabetes Mellitus Patients

**DOI:** 10.3390/ijms22105263

**Published:** 2021-05-17

**Authors:** Noha Mousaad Elemam, Hind Hasswan, Hayat Aljaibeji, Nabil Sulaiman

**Affiliations:** 1 Sharjah Institute for Medical Research, College of Medicine, University of Sharjah, Sharjah 27272, United Arab Emirates; noha.elemam211@gmail.com (N.M.E.); hind.smsm@gmail.com (H.H.); haljaibeji@bwh.harvard.edu (H.A.); 2 Department of Neurology, Brigham and Women’s Hospital, Boston, MA 02115, USA; 3 Department of Family Medicine, College of Medicine, University of Sharjah, Sharjah 27272, United Arab Emirates; 4 Baker/IDI Heart and Diabetes Institute, Melbourne, VIC 3004, Australia

**Keywords:** miRNAs, soluble ACE2, type 2 diabetes mellitus, biomarkers

## Abstract

The global coronavirus disease 2019 (COVID-19) pandemic was associated with multiple organ failure and comorbidities, such as type 2 diabetes mellitus (T2DM). Risk factors, such as age, gender, and obesity, were associated with COVID-19 infection. Severe acute respiratory syndrome coronavirus 2 (SARS-CoV-2) is known to use several host receptors for viral entry, such as angiotensin-converting enzyme 2 (ACE2) and transmembrane protease serine 2 (TMPRSS2) in the lung and other organs. However, ACE2 could be shed from the surface to be soluble ACE2 (sACE2) in the circulation. The epigenetic factors affecting ACE2 expression include a type of small non-coding RNAs called microRNAs (miRNAs). In this study, we aimed at exploring the status of the sACE2 as well as serum levels of several upstream novel miRNAs as non-invasive biomarkers that might have a potential role in T2DM patients. Serum samples were collected from 50 T2DM patients and 50 healthy controls, and sACE2 levels were quantified using enzyme-linked immunosorbent assay (ELISA). Also, RNA was extracted, and TaqMan miRNA reverse transcription quantitative PCR (RT-qPCR) was performed to measure serum miRNA levels. Our results revealed that sACE2 is decreased in the T2DM patients and is affected by age, gender, and obesity level. Additionally, 4 miRNAs, which are revealed by in silico analysis to be potentially upstream of ACE2 were detectable in the serum. Among them, miR-421 level was found to be decreased in the serum of diabetic patients, regardless of the presence or absence of diabetic complications, as well as being differential in various body mass index (BMI) groups. The other 3 miRNAs (miR-3909, miR-212-5p, and miR-4677-3p) showed associations with multiple factors including age, gender, BMI, and serum markers, in addition to being correlated to each other. In conclusion, our study reveals a decline in the circulating serum levels of sACE2 in T2DM patients and identified 4 novel miRNAs that were associated with T2DM, which are influenced by different clinical and demographic factors.

## 1. Introduction

It has been over a year since the discovery of severe acute respiratory syndrome coronavirus 2 (SARS-CoV-2), the virus responsible for coronavirus disease 2019 (COVID-19) infection, yet so much information remains a mystery. SARS-CoV-2 was found to affect multiple systems including the cardiac, renal, and digestive systems. Age, gender, and obesity were known risk factors associated with COVID-19 infection [1]. Diabetes mellitus was reported to be one of the most common comorbidities with COVID-19 infection [2,3,4,5,6]. Furthermore, diabetic patients were found to show more critical symptoms, higher ICU admission rates as well as higher mortalities [4]. This is particularly true in the Middle East region, specifically the United Arab Emirates (UAE), where the prevalence of diabetes is quite high [7]. 

Like SARS-CoV virus, SARS-CoV-2 is known to use several host receptors for viral entry, such as angiotensin-converting enzyme 2 (ACE2) and transmembrane protease serine 2 (TMPRSS2) [8,9,10,11,12]. It was reported that ACE2 expression is higher in the pancreas and lung tissues of type 2 diabetes mellitus (T2DM) patients, possibly explaining the increased risk of diabetic patients getting infected by SARS-CoV-2 [13,14]. Sometimes, ACE2 is shed from the surface using the metalloproteinase ADAM17 to be in a soluble form [15]; however, soluble ACE2 (sACE2) status in diabetes mellitus remains unknown. Previous studies shed the light on the effect of sACE2 in various infections, such as influenza A (H7N9) infection [16]. Additionally, serum levels of sACE2 were linked with clinical outcomes [16,17]. 

MicroRNAs (miRNAs) are small, single-stranded, non-coding RNA molecules that regulate gene expression at post-transcriptional and translational levels [18]. Several studies explored the interaction between miRNAs and SARS-CoV-2, which suggested potential therapeutic outcomes [19]. However, in COVID-19 patients with multifocal interstitial pneumonia, low serum levels of miRNAs related to the regulation of inflammation were associated with poor prognosis, high mortality rate, and subtherapeutic outcome [20]. On another note, miRNAs were explored in COVID-19 several organ complications. For instance, in rat and human cardiomyocytes, miR-200c was found to target ACE2 which indicated a potential preventive factor against COVID-19 cardiovascular complications [21,22]. The potential role of ACE2-related microRNAs in COVID-19 renal complications was also explored [23]. In fact, several miRNAs are found in the human serum where they show potential to be biomarker candidates [24,25,26]. Specifically, several miRNAs showed great potential to be biomarkers in type 1 diabetes mellitus (T1DM) and T2DM [27].

Hence, in this study, we aimed at investigating the serum levels of sACE2 and upstream miRNAs that have never been studied before in T2DM patients. This might shed the light on potential biomarkers for diabetes and possibly COVID-19 infection.

## 2. Results

### 2.1. Soluble ACE2 Is Lower in Diabetic Patients

**Figure 1:**
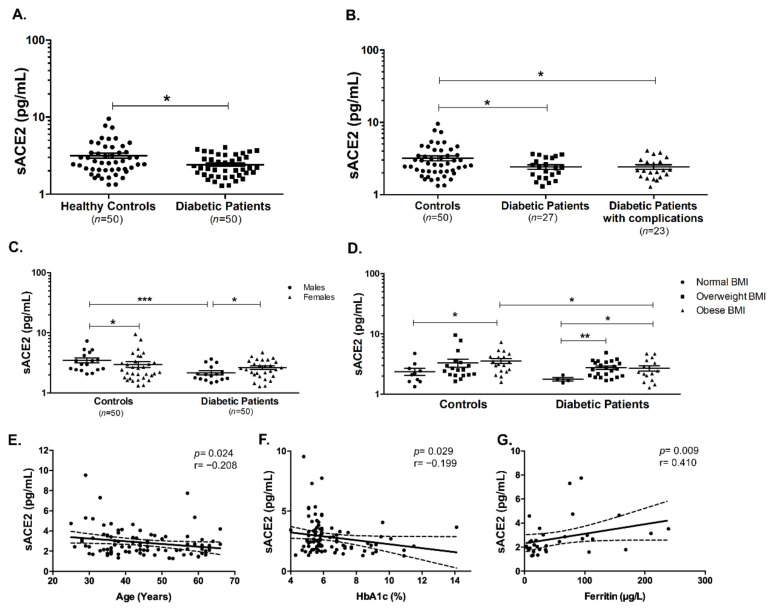
Serum levels of soluble ACE2 in diabetic patients and healthy controls. (**A**) sACE2 was significantly reduced in diabetic patients compared to healthy controls. (**B**) Decline in sACE2 concentration was significant in diabetic patients with the presence and absence of complications. sACE2 levels of healthy controls and diabetic patients were affected by (**C**) gender and (**D**) BMI. There were significant correlations between sACE2 and (**E**) age, (**F**) HbA1c, and (**G**) ferritin levels, respectively. Dashed lines represent 95% confidence intervals. * *p* < 0.05, ** *p* < 0.01, and *** *p* < 0.001.

It was interesting to investigate the level of shed sACE2 in the serum of T2DM patients. As shown in Figure 1A, sACE2 was decreased in the serum of T2DM patients (*p* < 0.05). Furthermore, it seemed to be downregulated in those patients with and without complications (*p* < 0.05 and *p* < 0.05, respectively, Figure 1B). Also, sACE2 was found to be significantly reduced in male diabetic patients compared to male healthy controls (*p* < 0.001). Interestingly, sACE2 was found to be higher in male healthy individuals compared to female controls; however, this was reversed in T2DM patients which showed lower sACE2 serum levels (*p* < 0.05, Figure 1C). 

Obesity was found to affect ACE2 expression on various cell types, and thus it was crucial to see if there was an effect of body mass index (BMI) on serum sACE2. As shown in Figure 1D, sACE2 was higher in obese healthy controls (*p* < 0.05), while there were significant elevations of the levels of sACE2 in overweight (*p* < 0.01) and obese (*p* < 0.05) diabetic patients. Additionally, there were significant negative correlations between sACE2 and age (Spearman’s r = −0.208, *p* = 0.024, Figure 1E) and between sACE2 and glycated hemoglobin (HbA1c) (Spearman’s r = −0.199, *p* = 0.029, Figure 1F), respectively. Moreover, there was a significant positive correlation between sACE2 and ferritin (Spearman’s r = 0.410, *p* = 0.009, Figure 1G).

### 2.2. In Silico Analysis Reveals 5 Potential miRNAs that Could Target ACE2

Bioinformatics analysis was done using 4 different tools (miRwalk, miRDB, TargetScan, and Diana Lab). Several miRNAs were suggested by each tool that could be possible upstream miRNAs of ACE2. In order to ensure the right selection for miRNAs to be further validated in vitro, selection criteria were implemented where only miRNAs identified by at least 3 of the 4 tools were shortlisted and chosen for validation. These included miR-421, miR-3909, miR-212-5p, miR-4677-3p, and miR-4766-5p, respectively. Among them, miR-421 was recognized by all 4 tools: miRDB, Diana Lab, TargetScan, and miRWalk, miR-212-5p and miR-4677-3p were suggested by miRDB, TargetScan, and miRWalk tools, whereas miR-3909 and miR-4766-5p were identified by miRDB, Diana Lab, and TargetScan tools. A literature search revealed that these 5 miRNAs were never investigated before in the serum of T2DM patients.

### 2.3. Serum Level of miR-421 Is Lower in T2DM Patients

**Figure 2:**
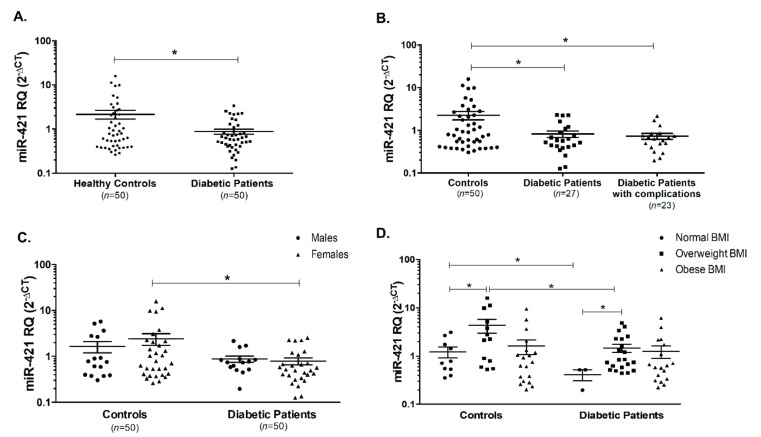
Serum levels of miR-421 in diabetic patients and healthy controls. (**A**) miR-421 was significantly reduced in diabetic patients compared to healthy controls. (**B**) Declines in miR-421 levels were significant in diabetic patients with the presence and absence of complications. (**C**) miR-421 was significantly reduced in female diabetic patients compared to female healthy controls. (**D**) miR-421 levels were significantly altered in the different categories of healthy controls and diabetic patients depending on BMI. * indicates *p* < 0.05.

miR-421 was assessed in the serum of T2DM patients and healthy controls, where it was found to be reduced in the diabetic patients when compared with the healthy controls, whether these patients are having complications or not (Figure 2A,B, *p* < 0.05). Moreover, upon gender classification of controls and patients, the same pattern of decline in miR-421 serum level was observed in female diabetic patients compared to healthy female individuals (Figure 2C). Interestingly, miR-421 showed an upregulation in overweight healthy controls compared to healthy controls with a normal BMI range (*p* < 0.05, Figure 2D). A similar pattern was observed in the overweight diabetic patients (*p* < 0.05, Figure 2D). Finally, miR-421 was lower in the diabetic patients whether they have a normal BMI range or an overweight range in comparison to the respective groups in the healthy controls (*p* < 0.05, Figure 2D).

### 2.4. Serum Levels of miR-3909, miR-212-5p, and miR-4677-3p Remain Unchanged in T2DM Patients

**Figure 3:**
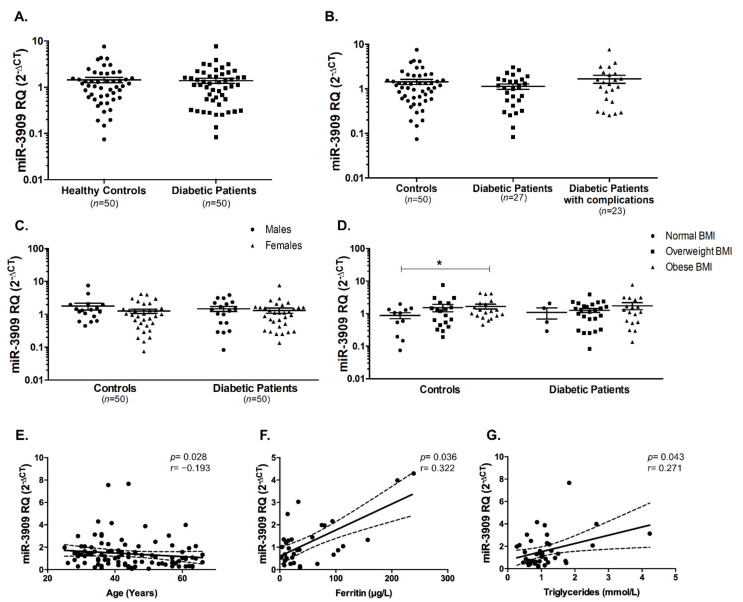
miR-3909 was unaltered in T2DM patients compared to healthy controls. (**A**) miR-3909 was not changed in all T2DM patients as well as (**B**) in diabetic patients with or without diabetic complications. (**C**) miR-3909 levels were unaffected by gender in healthy controls and diabetic patients. (**D**) miR-3909 was elevated in obese healthy individuals compared to healthy individuals with normal BMI. There was a significant (**E**) negative correlation between miR-3909 and age, and there were positive correlations between miR-3909 and (**F**) ferritin, and (**G**) triglycerides, respectively. Dashed lines represent 95% confidence intervals. * indicates *p* < 0.05.

Another possible upstream miRNA is miR-3909, which was investigated in diabetic patients and healthy controls. As illustrated in Figure 3A, it was not significantly changed in T2DM patients. Furthermore, diabetic patients with or without complications did not show a differential expression of miR-3909 (Figure 3B). Also, there was no difference in miR-3909 expression in males compared to females (Figure 3C). Upon dividing the healthy controls and T2DM patients into different BMI categories, there was a significant increase in miR-3909 in obese healthy controls compared to the respective normal BMI healthy individuals (*p* < 0.05, Figure 3D). Interestingly, we observed a significant negative correlation between miR-3909 and age (Spearman’s r = −0.193, *p* = 0.028, Figure 3E), while there was a positive correlation between miR-3909 and ferritin (Spearman’s r = 0.322, *p* = 0.036, Figure 3F), and a significant positive correlation between miR-3909 and triglycerides (TGs) (Spearman’s r = 0.271, *p* = 0.043, Figure 3G).

**Figure 4:**
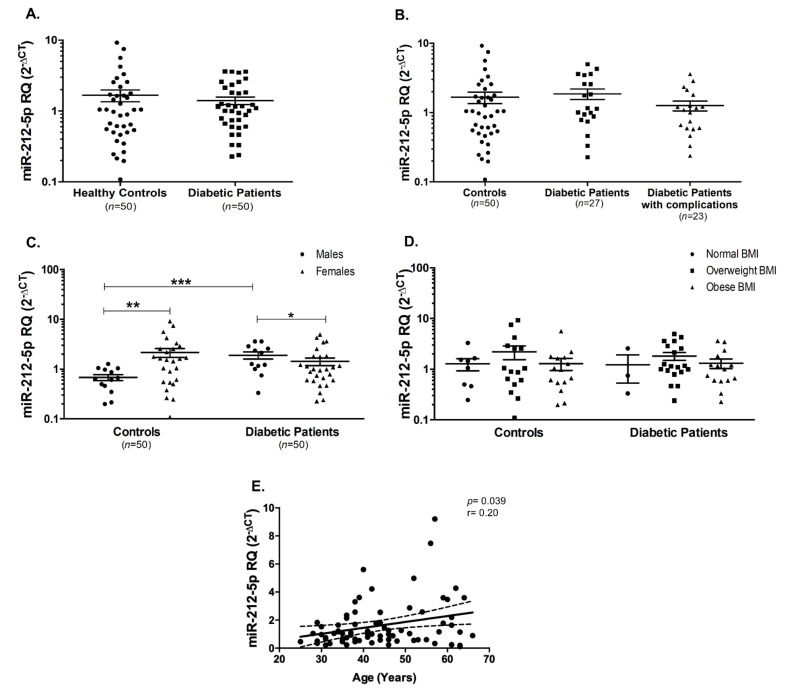
miR-212-5p was unaltered in T2DM patients compared to healthy controls. (**A**) miR-212-5p was not changed in all T2DM patients as well as (**B**) in diabetic patients with or without diabetic complications. (**C**) miR-212-5p levels were significantly altered by gender in healthy controls and diabetic patients. (**D**) miR-212-5p was unaffected in the different categories of healthy controls and diabetic patients depending on BMI. (**E**) There was a significant positive correlation between miR-212-5p and age. Dashed lines represent 95% confidence intervals. * *p* < 0.05, ** *p* < 0.01, and *** *p* < 0.001.

Similarly, miR-212-5p serum levels did not change in T2DM patients compared to healthy controls (Figure 4A), as well as in diabetic patients with or without complications (Figure 4B). In healthy controls, miR-212-5p was higher in the females compared to the males (*p* < 0.01, Figure 4C). Interestingly, an opposite pattern was observed in the diabetic patients (*p* < 0.05, Figure 4C). Furthermore, miR-212-5p seems to be significantly elevated in male diabetic patients compared to male healthy controls (*p* < 0.001, Figure 4C). miR-212-5p did not show any significant changes among different BMI groups, whether in healthy controls or in diabetic patients (Figure 4D). On the other hand, there was a significant positive correlation between miR-212-5p and age (Spearman’s r = 0.20, *p* = 0.039, Figure 4E). 

**Figure 5:**
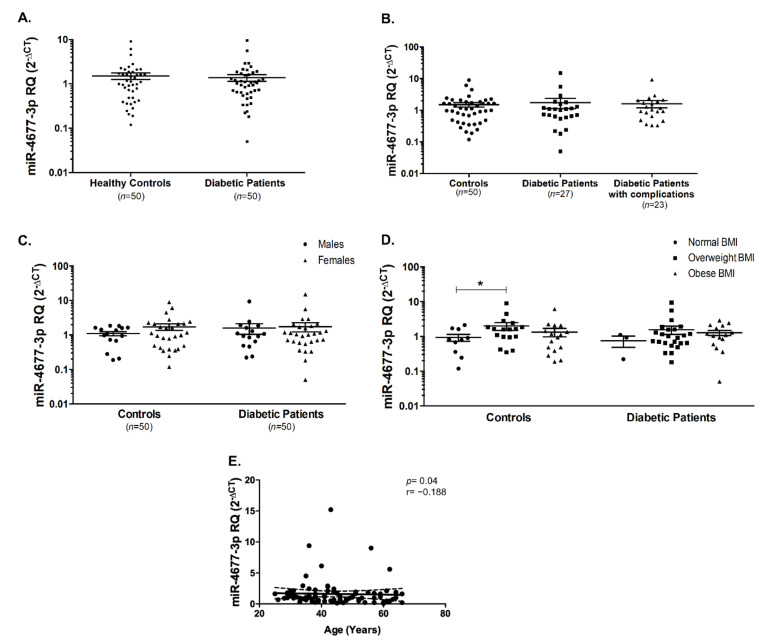
miR-4677-3p was unaltered in T2DM patients compared to healthy controls. (**A**) miR-4677-3p was not changed in all T2DM patients as well as (**B**) in diabetic patients with or without diabetic complications. (**C**) miR-4677-3p levels were unaltered by gender in healthy controls and diabetic patients. (**D**) miR-4677-3p was elevated in healthy overweight individuals compared to healthy individuals with normal BMI. (**E**) There was a negative correlation between miR-4677-3p and age. Dashed lines represent 95% confidence intervals. * indicates *p* < 0.05.

miR-4677-3p did not show any differential expression in diabetic patients and healthy controls (Figure 5A), as well as not being affected by the presence or absence of diabetic complications (Figure 5B). Moreover, miR-4677-3p did not seem to be affected by gender or BMI, except for being significantly elevated (*p* < 0.05) in overweight healthy controls compared to those with normal BMI (Figure 5C,D). However, there was a significant negative correlation between miR-4677-3p and age (Spearman’s r = −0.188, *p* = 0.04, Figure 5E). It is noteworthy to report that the fifth investigated miRNA, miR-4766-5p was undetected in the serum of healthy controls and diabetic patients.

**Figure 6:**
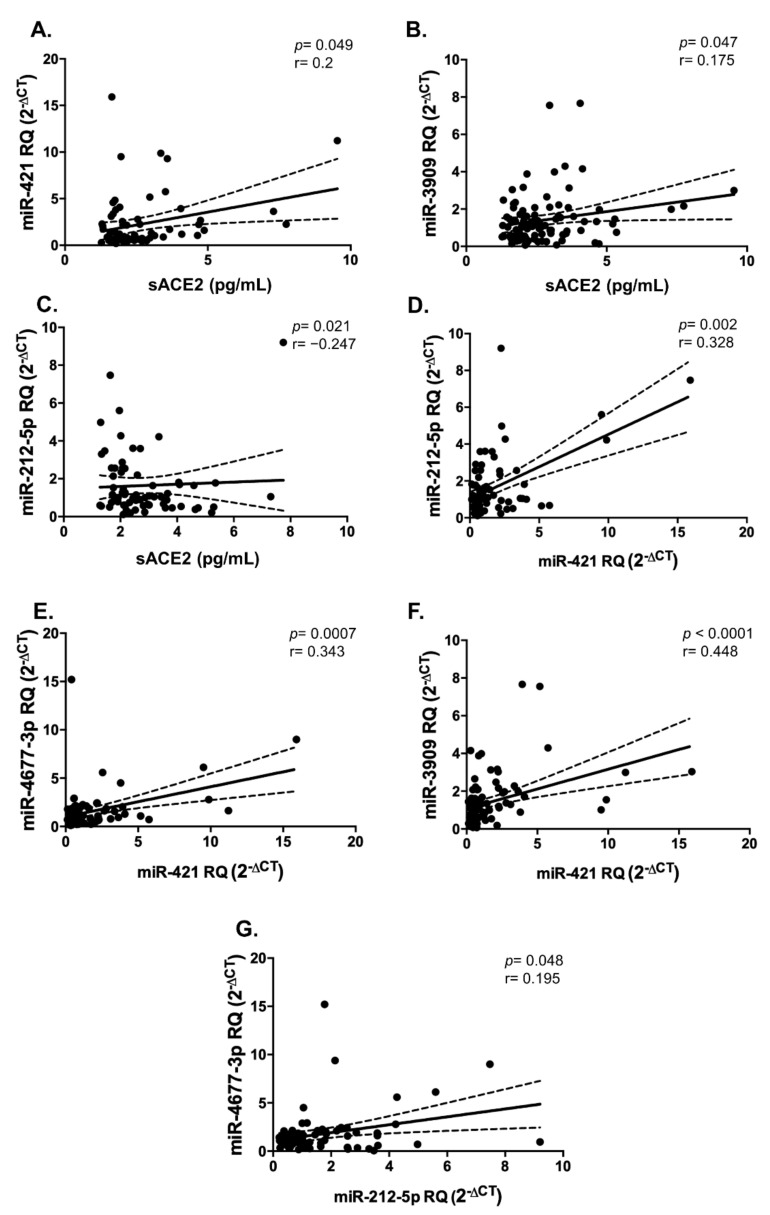
Correlation analyses between sACE2 and 4 serum miRNAs. There were significant positive correlations between (**A**) miR-421 and sACE2, (**B**) miR-3909 and sACE2, and a significant negative correlation between (**C**) miR-212-5p and sACE2, respectively. Also, there was a positive correlation between (**D**) miR-212-5p and miR-421, (**E**) miR-4677-3p and miR-421, (**F**) miR-3909 and miR-421, and (**G**) miR-4677-3p and miR-212-5p, respectively. Dashed lines represent 95% confidence intervals.

It was quite interesting to assess if sACE2 and the four investigated miRNAs are correlated to each other. Indeed, sACE2 was found to be significantly positively correlated with miR-421 (Spearman’s r = 0.2, *p* = 0.049, Figure 6A), and miR-3909 (Spearman’s r = 0.175, *p* = 0.047, Figure 6B), while being negatively correlated with miR-212-5p (Spearman’s r = −0.247, *p* = 0.021, Figure 6C). In addition, miR-421 was found to show a significant positive correlation with the other miRNAs: miR-212-5p (Spearman’s r = 0.328, *p* = 0.002, Figure 6D), miR-4677-3p (Spearman’s r = 0.343, *p* = 0.0007, Figure 6E), and miR-3909 (Spearman’s r = 0.448, *p* < 0.0001, Figure 6F). Further, miR-4677-3p showed a positive significant correlation with miR-212-5p (Spearman’s r = 0.195, *p* = 0.048, Figure 6G).

## 3. Discussion

Little information is known about the COVID-19 infection. Researchers around the globe are still investigating the different factors and comorbidities that are associated with COVID-19 infection. Since this pandemic’s announcement, diabetes mellitus was considered one of the most common comorbidities with COVID-19 infection, where diabetic patients presented severe complications and higher mortality [2,3,4,5,6,28]. Upon shedding of ACE2 by ADAM17 from the cell surface, it can be found in a soluble form circulating in the serum/plasma of individuals [15]. Soluble ACE2 (sACE2) status was previously investigated in various diseases, such as influenza A infection and cardiac dysfunction [16,29,30]. Moreover, sACE2 was previously explored in the serum of patients with T1DM, it was increased especially in male T1DM patients compared to male controls [31]. However, sACE2 was not investigated before in T2DM. In this study, we found that sACE2 is decreased in the serum of T2DM patients, with or without any complications associated with diabetes, such as retinopathy, nephropathy, or neuropathy. This implies that there could be a decrease in the shedding of surface ACE2 in T2DM patients, as previously suggested by the previous study reporting a higher ACE2 expression in the pancreas and lungs of diabetic patients [13,14]. Circulating sACE2 was previously reported to play a protective role from viral infections, such as influenza A [16]. Hence, the observed decrease in sACE2 in diabetic patients might further explain the rationale behind their increased susceptibility to SARS-CoV-2 infection. Another crucial finding was the differential sACE2 levels in different gender and BMI groups. In healthy controls, sACE2 was found to be higher in males than females, whereas a reverse pattern was observed in diabetic patients. This was similar to the several previous studies where plasma sACE2 levels were reported to be higher in men than in women [32,33,34]. On the other hand, the observed increase in sACE2 levels of female diabetic patients compared to male diabetic patients, showed a similar pattern to those reported by previous studies that showed a more pronounced increase in circulating ACE2 in human diabetic females and rats [35,36]. Furthermore, our findings are in agreement with the results obtained in previous literatures showing that sACE2 is associated with BMI and obesity as well as age [32,34,37]. Another indicator of the potential role of sACE2 as a biomarker in T2DM patients is the correlation between HbA1c and sACE2. 

Besides being an iron sequester protein, ferritin is known to be a proinflammatory cytokine that is associated with inflammation, such as that observed in infections [38,39,40]. Recent work indicated that critically ill COVID-19 patients had marked elevations of serum levels of ACE2, ferritin, and IL-6 [41,42]. Serum ferritin levels were strongly linked to the severity of COVID-19 [43,44]. Interestingly, our data showed that ferritin exhibited a positive correlation with sACE2, indicating that sACE2 might show a robust potential in inflammation associated with diseases including diabetes and COVID-19.

Another group of potential biomarkers is the non-coding RNA molecules called miRNAs [24,25,26]. Several miRNAs showed great potential to be biomarkers in T1DM and T2DM [27]. In silico analysis revealed 5 novel miRNAs to be upstream of ACE2, by at least 3 out of the 4 different used tools. Moreover, these miRNAs were not investigated before in the serum of T2DM patients. A recent study by Hum C. et al. used bioinformatics software to identify candidate miRNAs that target the 3′-untranslated region (UTR) of ACE2 in humans. These miRNAs included miR-362-5p, miR-421, miR-500a-5p, miR-500b-5p, miR-3909, and miR-4766-5p, respectively [19], where 3 of these miRNAs were similarly identified in our study.

We observed a decline in the level of miR-421 in diabetic patients compared to healthy controls, especially in females and irrespective of the presence or absence of diabetic complications. Furthermore, the reduction in miR-421 was reported in diabetic patients with normal and overweight BMI range. Upon classification of healthy controls and diabetic patients based on gender, we found that there was no significant difference in miR-421 levels between males and females. This finding is concordant with a study by Li Y. et al. where no relationship was found between miR-421 expression and age or gender in lung cancer patients [45]. Interestingly, circulating miR-421 was found to be increased in individuals with BMI in the overweight range, whether they belong to controls’ or diabetic patients’ group. This is the first study to link the expression of circulating miR-421 and obesity. Intriguingly, miR-421 was positively correlated with sACE2, and all other detected 3 miRNAs indicating that they could possibly be associated with each other. This goes in line with a previous study highlighting the role of miR-421 in regulating ACE2 expression, with was further validated using in vitro reporter luciferase assay [46]. Only one of the investigated miRNAs, miR4766-5p was not detected in the serum of all included participants.

Two of the investigated miRNAs, miR-3909 and miR-4677-3p, were detected in the serum but their levels were not different in the serum of diabetic patients compared to healthy controls. Also, miR-3909 and miR-4677-3p seem to be unaffected by the presence/absence of diabetic complications or gender. However, they both seem to have a link to obesity. For instance, miR-3909 was found to be slightly increased in the overweight group, while it was significantly increased in the obese group of healthy controls. This was further emphasized by the positive correlation between miR-3909 and triglycerides in all of the included participants. This goes in line with another study by Kuryłowicz A. et al. where miR-3909 was among the differentially expressed miRNAs associated with obesity [47]. The correlation between miR-3909 and sACE2 showed a positive correlation in the included subjects, whether they are diabetic patients or healthy controls. On another note, miR-4677-3p was significantly increased in overweight healthy individuals, while there was a slight increase in the diabetic overweight patients. 

Lastly, miR-212-5p was found to be not significantly changed between all the diabetic patients and healthy controls. However, upon gender stratification of the subjects, it seemed that serum levels of miR-212-5p were increased in the male diabetic patients compared to male healthy controls. Furthermore, the serum levels of miR-212-5p were higher in female healthy controls compared to male healthy controls, indicating that this miRNA expression could be affected by gender. This finding differed from previous observations that miR-212 serum levels were not statistically significantly associated with gender [48]. Regarding age, we found that the serum level of miR-212-5p was positively correlated with age. Similarly, previous studies showed that miR-212-5p was linked to age-regulated transcriptome as well age-associated diseases, such as late-onset Alzheimer’s disease [49,50].

In conclusion, our study reveals a decrease in the circulating serum levels of sACE2 in patients with type 2 diabetes, highlighting a possible rationale for their higher susceptibility to COVID-19 infection. Furthermore, serum levels of 4 novel miRNAs, upstream of ACE2, were reported to be affected by age, gender, BMI, and diabetes which could be further investigated to possibly understand their cellular mechanism of action by altering ACE2 expression. However, our study has several limitations including that the sample size is relatively small and only T2DM was studied. Future studies could further examine the expression levels of these miRNAs and ACE2 in the cellular players of diabetes, such as pancreatic islets. Additionally, such an investigation might include the assessment of soluble and cellular enzymes, such as ADAM17, that shed surface ACE2 into the circulation.

## 4. Subjects and Methods

### 4.1. Recruitment of Controls and Patients

A total of 100 subjects were recruited in this study, including 50 healthy controls and 50 T2DM patients. All the subjects included in this study were of Emirati nationality and were selected from All-New Diabetes in Sharjah and Ajman (ANDISA) project, in which patients and controls were recruited to the study based on their routine visits to the endocrinology clinic at the University Hospital of Sharjah. 

The groups of healthy controls and T2DM patients were age, gender, and BMI matched. All the characteristics were extracted from the study subjects’ clinical records. The included T2DM patients did not have hypertension as comorbidity and did not take any class of ACE inhibitors including Angiotensin II receptor blockers (ARBs). Some T2DM patients had diabetic complications, including nephropathy, neuropathy, and retinopathy. The inclusion criteria included Emirati individuals with or without diabetes in the presence or absence of associated complications of T2DM, while exclusion was done for pre-diabetic individuals. Collected data included age, gender, BMI, fasting blood glucose levels, HbA1c, high-density lipoprotein cholesterol (HDL-C), low-density lipoprotein cholesterol (LDL-C), triglycerides (TGs), C-reactive protein (CRP), ferritin, and iron. Table 1 shows the demographic, clinical, and biochemical data of diabetic patients and healthy controls. The ethical approval was obtained by the UAE Ministry of Health (MOHAP/DXB/SUBC/No.14/2017, 30 April 2017) as well as the University of Sharjah (UOS) ethical committee (4 October 2017). All participants signed an informed consent form before blood sample and data collection.

### 4.2. In Silico Analysis for Upstream miRNAs that Target ACE2

Bioinformatics analysis using 4 tools was performed to identify miRNAs that target ACE2 mRNA. These tools included miRDB (http://mirdb.org/, accessed on 3 July 2020), Diana Lab (https://www.dianalab.gr/, accessed on 3 July 2020), TargetScan Human (http://www.targetscan.org/vert_72/, accessed on 3 July 2020), and miRWalk (http://mirwalk.umm.uni-heidelberg.de/, accessed on 3 July 2020). 

### 4.3. Serum Collection and RNA Extraction

After collection of 4 mL of whole blood in EDTA collection tube, samples were centrifuged to collect the serum that was stored at −80 °C until further use. 200 μls of serum were used for extraction of miRNAs, using miRNeasy Serum/Plasma Kit (Qiagen, Hilden, Germany), as per manufacturer’s protocol. The final elution was done in 20 μls of nuclease-free water. 

### 4.4. Human Serum miRNA Quantification

Specific cDNA for each of the respective miRNAs was obtained using High-Capacity cDNA Reverse Transcription Kit (Thermo Fisher Scientific, Waltham, MA, USA) and TaqMan MicroRNA Assay reagents (Applied Biosystems, Foster City, CA, USA) for each of the 5 miRNAs: miR-421, miR-212-5p, miR-3909, miR-4677-3p, and miR-4766-5p.

The miRNA expression levels were quantified using QuantStudio 3 Real-time qPCR (Applied Biosystems, Foster City, CA, USA) along with 5× HOT FIREPol Probe Universal qPCR Mix (SolisBioDyne, Tartu, Estonia) and the respective target primers: FAM tagged hsa-miR-421 (Assay ID: 002700), mmu-miR-212-5p (Assay ID: 461768_mat), hsa-miR-3909 (Assay ID: 465180_mat), hsa-miR-4677-3p (Assay ID: 465015_mat), and hsa-miR-4766-5p (Assay ID: 463003_mat). 

### 4.5. Enzyme-Linked Immunosorbent Assay (ELISA) Quantification of Soluble ACE2 (sACE2)

The sACE2 was measured in the serum of healthy controls and T2DM patients using the Human ACE-2 DuoSet ELISA (R&D Systems, Minneapolis, MN, USA), according to the manufacturer’s instructions. Concentration was calculated in pg/mL. 

### 4.6. Statistical Analysis

For the calculation of serum miRNA levels, the relative quantification was calculated using the equation (RQ = 2^−Δ*C*t^), where the Δ*C*t is calculated based on the average Ct values of the healthy controls, as previously described [51,52]. Statistical analyses were done using GraphPad Prism 6 (GraphPad Software, San Diego, CA, USA). All the variables are expressed as mean ± standard error of the mean (SEM). The data were subjected to normality tests, after which the data were compared using the Mann–Whitney U-test, while the correlation analysis was performed using Spearman’s method. *p* < 0.05 was considered statistically significant. 

## Figures and Tables

**Table 1:** Demographic, clinical, and biochemical characteristics of healthy controls and T2DM patients. Abbreviations: BMI, body mass index; CRP, C-reactive protein; FBG, fasting blood glucose; HDL-C, high-density lipoprotein cholesterol; LDL-C, low-density lipoprotein cholesterol; T2DM, type 2 diabates mellitus; TGs, triglycerides.

	Healthy Controls (*n* = 50)	T2DM Patients (*n* = 50)
Complications	-	No complications (*n* = 27)
		Neuropathy (*n* = 6), Retinopathy (*n* = 7), Nephropathy (*n* = 2), Macrovascular (*n* = 1), More than one complication (*n* = 7)
Gender (M/F)	18/32	18/32
Age (Years)	44.16 ± 12.19	44.36 ± 10.20
BMI (kg/m^2^)	29.27 ± 6.11	29.60 ± 3.91
HbA1c (%)	5.42 ± 0.43	7.49 ± 1.97
FBG (mmol/L)	5.45 ± 0.42	7.72 ± 3.21
HDL-C (mmol/L)	1.66 ± 0.53	1.45 ± 1.14
LDL-C (mmol/L)	2.91 ± 0.63	3.23 ± 1.12
TGs (mmol/L)	0.91 ± 0.49	1.47 ± 0.87
CRP (mg/L)	8.07 ± 15.09	18.21 ± 16.66
Ferritin (µg/L)	63.38 ± 66.17	43.80 ± 47.62
Iron (µmol/L)	13.13 ± 5.87	7.73 ± 3.48
Height (cm)	161.57 ± 8.91	164.09 ± 9.75
Weight (kg)	75.64 ± 16.40	79.94 ± 13.16
